# A new NLR disease resistance gene *Xa47* confers durable and broad-spectrum resistance to bacterial blight in rice

**DOI:** 10.3389/fpls.2022.1037901

**Published:** 2022-11-24

**Authors:** Yuanda Lu, Qiaofang Zhong, Suqin Xiao, Bo Wang, Xue Ke, Yun Zhang, Fuyou Yin, Dunyu Zhang, Cong Jiang, Li Liu, Jinlu Li, Tengqiong Yu, Lingxian Wang, Zaiquan Cheng, Ling Chen

**Affiliations:** ^1^ Biotechnology and Germplasm Resources Institute, Yunnan Academy of Agricultural Sciences, Yunnan Provincial Key Lab of Agricultural Biotechnology, Kunming, China; ^2^ College of Plant Protection, Yunnan Agricultural University, Kunming, China

**Keywords:** *Xa47*, NLR, *Xanthomonas oryzae* pv. *oryzae*, broad-spectrum resistance, bacterial blight

## Abstract

Bacterial blight (BB) induced by *Xanthomonas oryzae* pv. *oryzae* (Xoo) is a devastating bacterial disease in rice. The use of disease resistance (*R*) genes is the most efficient method to control BB. Members of the nucleotide-binding domain and leucine-rich repeat containing protein (NLR) family have significant roles in plant defense. In this study, *Xa47*, a new bacterial blight *R* gene encoding a typical NLR, was isolated from G252 rice material, and XA47 was localized in the nucleus and cytoplasm. Among 180 rice materials tested, *Xa47* was discovered in certain BB-resistant materials. Compared with the wild-type G252, the knockout mutants of *Xa47* was more susceptible to Xoo. By contrast, overexpression of *Xa47* in the susceptible rice material JG30 increased BB resistance. The findings indicate that *Xa47* positively regulates the Xoo stress response. Consequently, *Xa47* may have application potential in the genetic improvement of plant disease resistance. The molecular mechanism of *Xa47* regulation merits additional examination.

## Introduction

Crop production occurs in complex and changing ecological environments, and plants inevitably experience many biotic stresses, including attacks by fungi, bacteria, viruses, and insects (Savary et al., 2019; Zhang et al., 2020). To prevent onset of disease, plants evolved innate immune mechanisms to stop the invasion of pathogens primarily by recognizing pathogenic signals by various genes and associated gene products (Ahuja et al., 2012; Bednarek, 2012). When a pathogen invades, the first level of a plant defense system is activated, and pattern recognition receptors on plant cell membranes recognize pathogen/microbe-associated molecular patterns (PAMPs/MAMP), triggering PAMPs/MAMPs-induced immunity (PTI/MTI), which inhibits further pathogen spread (Tena et al., 2011). To interfere with or break plant PTI/MTI, pathogens secrete effectors or virulence factors that enter plant cells directly or indirectly to cause infection (Chen and Ronald, 2011). However, because of co-evolution with pathogens, the effectors can be recognized directly or indirectly by plant resistance proteins to trigger effector-triggered immunity (ETI), a second level of a plant immune system, which is also known as “gene-to-gene” disease resistance (Jones and Dangl, 2006; Dodds and Rathjen, 2010). Activation of ETI is usually accompanied by a burst of reactive oxygen species and a hypersensitive response (HR), among other actions, to prevent pathogen colonization (Spoel and Dong, 2012; Withers and Dong, 2017).

Rice is one of the most important agricultural crops and is consumed by more than half of the global population (Ainsworth, 2008). Rice production is often threatened by pathogens, and rice leaf blight (BB) caused by *Xanthomonas oryzae* pv. *oryzae* (Xoo) can cause 10% to 30% reductions in yield or even crop failure (Liu et al., 2014). Compared with chemical applications, cultivating resistant varieties is a more effective and environmentally friendly method to control BB in rice (Ke et al., 2017; Li et al., 2020; Zhang et al., 2020). The interaction between rice and Xoo has become an important model in analyzing plant disease resistance and bacterial pathogens (Niño-Liu et al., 2006). Clarifying the various molecular mechanisms of this interaction is of great importance to basic research and crop breeding (Jiang et al., 2020). To date, 12 genes against Xoo have been isolated: *Xa1*, *Xa3/Xa26*, *Xa4*, *xa5*, *Xa7*, *Xa10*, *xa13*, *Xa21*, *Xa23*, *xa25*, *Xa27*, and *xa41* (Song et al., 1995; Yoshimura et al., 1998; Iyer and McCouch, 2004; Sun et al., 2004; Gu et al., 2005; Jiang et al., 2006; Xiang et al., 2006; Yang et al., 2006; Liu et al., 2011; Tian et al., 2014; Wang et al., 2015; Hutin et al., 2016; Hu et al., 2017; Chen et al., 2021). The genes encode multiple protein types and are an important in the rice–Xoo interaction. Notably, nine of the genes (*Xa1*, *Xa7*, *Xa10*, *Xa23*, *Xa27*, *xa5*, *xa13*, *xa25*, and *xa41*) are related to the transcription activator-like effectors (TALEs) secreted by the type III secretion system (T3SS) of pathogens in the resistance mechanism of host cells (Boch and Bonas, 2010; Jiang et al., 2020).

The TALEs are important pathogenic factors secreted by Xoo that enter the host nucleus with the assistance of T3SS and then bind to a specifically identified target gene promoters to regulate the transcription of downstream genes (Bogdanove et al., 2010). The promoter regions that bind to TALEs are effector binding elements (EBEs). The target genes to which TALEs bind can be either susceptibility genes that regulate host plant physiology to help a pathogen survive and increase colonization or disease resistance genes that mediate the production of plant ETI (Boch et al., 2014; Peng et al., 2019). For example, instance, the expression of the rice executor resistance (*R*) genes *Xa7*, *Xa10*, *Xa23*, and *Xa27* leads to transcriptional activation by the TALEs AvrXa7/PthXo3, AvrXa10, AvrXa23, and AvrXa27, respectively, triggering a host-induced HR response to prevent Xoo colonization in rice and achieve strong disease resistance (Luo et al., 2021). Alternatively, Xoo TALEs (PthXo1, PthXo2, AvrXa7/Tal5/PthXo3/TalC) can directly target EBEs of the corresponding *OsSWEET* genes (*Xa13/OsSWEET11/Os8N3*, *Xa25/OsSWEET13*, and *Xa41/OsSWEET14/Os11N3*), thereby up-regulating the expression in rice, which supplies sufficient sugar for the growth of Xoo in host cells and increases susceptibility to BB (Yuan et al., 2009; Streubel et al., 2013; Zhou et al., 2015). Moreover, mutations in the EBEs of *xa13*, *xa25*, and *xa41* interfere with Xoo TALE interactions, resulting in cryptic resistance to BB (Chu et al., 2006; Yang et al., 2006; Liu et al., 2011; Yuan et al., 2010; Hutin et al., 2015; Zhou et al., 2015). In addition, *Xa1* and its alleles (*Xa2*, *Xa14*, *Xa31*, and *Xa45*) encode atypical nucleotide binding site-leucine-rich repeat (NBS-LRR) containing proteins that recognize multiple Xoo TALEs (PthXo1, Tal4, and Tal9d) and can confer resistance to BB in rice (Zhang et al., 2020). However, interfering TALEs (iTALEs), which are truncated to lack a transcriptional activation domain, can interfere with XA1, XA2, XA14, and XA45 to confer resistance to Xoo in rice (Zhang et al., 2020).

Previously, the gene *Xa47* was localized in a 26.24-kb interval between molecular markers R13I14 and 13rbq-71 and was found to co-segregate with the InDel marker Hxjy-1 (Xing et al., 2021). Based on analyses of the Gramene database and the rice genome RGAP annotation database, *LOC_Os11g46200* (in this study, *xa47*) was ultimately selected as the target gene of *Xa47* (Xing et al., 2021). The gene *Xa47* was originally discovered in the Yuanjiang common wild rice infiltration line material G252, but the gene has not been cloned. However, whether G252, a broad-spectrum, highly resistant rice germplasm material, is endowed with the resistance function of *Xa47* is unknown. Therefore, in this study, the *Xa47* gene was cloned, its function in improving resistance to BB was investigated, and its potential mechanism of resistance was examined.

## Materials and methods

### Plant materials and bacterial inoculation

The rice G252, a BC_2_F_16_ generation material of the Yuanjiang common wild rice infiltration line (IL), was the donor material for *Xa47* cloning and gene editing. Indica varieties susceptible to Xoo strains PXO99 and PB included IR24,IRBB1, and IRBB14. Dr. Zaiquan Cheng (Biotechnology & Genetic Germplasm Institute, Yunnan Academy of Agricultural Sciences, Yunnan, China) kindly provided 80 rice landraces and 100 ILs. Transgenic plants were grown in an artificial climate chamber at 28°C for 12 h (light) and 20°C for 12 h (dark) with relative humidity of 65% to 80%. The other rice materials were grown in the field.

The Xoo strains included the Chinese strains YN18 (C1), YN1 (C2), GD414 (C3), HEN11 (C4), ScYc-b (C5), YN7 (C6), JS49-6 (C7), FuJ (C8), and YN24 (C9), the Filipino strain PXO99, and PB, a strain with Tal3a and Tal3b genes knocked out from PXO99 (Ji et al., 2016). All Xoo strains were grown at 28°C on nutrient agar medium (1 g/L yeast extract, 12 g/L sucrose, 18 g/L agar, 5 g/L peptone, 3 g/L beef extract). Cultured Xoo strains were eluted with sterile water, and concentrations of bacterial suspensions were configured to 3 × 10^8^ colony forming units/mL (OD600 = 0.5). Rice plants at the gestation stage were inoculated with a Xoo strain using the sword leaf tip-clipping method. Disease lesion length (resistant: ≤6 cm; susceptible: >6 cm) was measured approximately 14 d after inoculation when disease development of the susceptible control material JG30 stabilized, as described previously (Chen et al., 2008). Each Xoo strain was tested in three replicates, each with three rice plants. A detailed description of the Xoo strains involved in this study can be found in **Supplementary Table 1**.

### Cloning and sequence analysis of *Xa47*

The CTAB method was used to extract rice genomic DNA (Porebski et al., 1997). Based on the sequence of *LOC_Os11g46200* (*xa47*), the primer pair Xa47-12F/R, with forward primer 5’-TGGTGCCTATACCTTCATTG-3’ and reverse primer 5’-AATTCGTCATGTTCTACTAGC-3’, was designed and used to clone the gene. PCR amplification of *Xa47* was performed using primers Xa47-12F/R and G252 genomic DNA, and the PCR products were used for sequencing. Based on the sequencing results, the CDS sequence of *Xa47* was obtained using SnapGene 6.0 software (GSL Biotech, USA). BLASTP^1^ (https://blast.ncbi.nlm.nih.gov/Blast.cgi) was used for the *Xa47* homologous gene search, and CD-search (https://www.ncbi.nlm.nih.gov/Structure/cdd/wrpsb.cgi) was used to predict protein structural domains. Multiple sequence alignment and phylogenetic analysis was performed using DNAMAN8.0 (Lynnon Biosoft, USA) and (Kumar et al., 2016), and bootstrap testing was performed with 500 replicates using the neighbor-joining method.

### Detection of *Xa47* in different germplasm resources

The gene *Xa47* was detected in 180 germplasm resources (**Supplementary Tables 2, 3**) using a molecular marker screening method combined with homologous cloning, in which the primers used were Hxjy-1F/R and Xa47-12F/R, in that order. In parallel, the rice materials were evaluated for resistance to strains C5 and C9 (described above).

### Subcellular localization of XA47

An Eastep^®^ Super Total RNA Isolation kit (Proegal, Shanghai, China) was used to extract total RNA from rice leaves. A HiScript^®^ III RT SuperMix for qPCR kit (Vazyme Biotech Co., Ltd., Nanjing, China) was used to synthesize cDNA. Based on the *Xa47* gene sequence and the pBE221-GFP expression vector, primers with double restriction enzyme sites XbaI and SalI were designed to amplify the *Xa47* CDS sequence without a terminator. Polyethylene glycol mediated the transient expression of the XA47::GFP and pBE221-GFP constructs in rice protoplasts under the control of the 35S promoter. Sixteen hours after inoculation, resulting protoplasts were observed using a laser scanning confocal microscope (Nikon A1, Tokyo, Japan).

### CRISPR-Cas9-based gene editing in rice

The target vector pH-ubi-cas9 and the entry vector pOs-sgRNA were generously provided by Dr. Liang Chen (College of Life Sciences, Xiamen University, China). The selection and design of sgRNA target sequences were based on the web tool CRISPR-P2.0 (http://crispr.hzau.edu.cn/CRISPR2/news.php) (He et al., 2021). The pOs-*Xa47*-sgRNA was obtained by ligation reaction of sgRNA with BsaI-digested pOs-sgRNA vector by T4 DNA Ligase (New England Biolabs Ltd., Beijing, China). Similarly, the *Xa47*-ubi-Cas9 recombinant vector was obtained by ligation reaction of pOs-*Xa47*-sgRNA vector and pH-ubi-cas9 vector by T4 Polynucleotide Kinase (New England Biolabs Ltd., Beijing, China). The *Xa47*-ubi-Cas9 vector was introduced into G252 by an *Agrobacterium*-mediated method (Zhang et al., 2016). All CRISPR plants were genotyped, target locus regions were amplified, and PCR products were Sanger sequenced.

### 35S::*Xa47* expression vector construction and transformation

The *Xa47*-CDS containing the homologous sequence of pCAMBIA1305 was amplified using the appropriate double digestion sites NcoII and BstEI based on the CDS sequence of *Xa47* and the pCAMBIA1305 expression vector. This approach used the primers *Xa47*-CDS-F/R and Xa47-35S-F/R (attached). The *Xa47* was ligated to the pCAMBIA1305 vector in the presence of T4 DNA Ligase (TaKaRa, Dalian, China), resulting in the CaMV35S promoter-driven 35S::Xa47. An *Agrobacterium*-mediated approach was used to deliver the 35S::*Xa47* expression vector into the indica rice variety JG30, which is sensitive to Xoo strains. All transgenic plants were screened for thaumatin resistance. The resistant transgenic plants were planted in greenhouse until maturity. The continuous PCR test for *Xa47* was carried out. T_2_ homozygous transgenic plants were selected for further analysis.

### Gene expression assays

From the inverted second leaves of wild-type and mutant lines (described above), RNA was isolated, and cDNA was synthesized. Reverse transcription quantitative PCR (RT-qPCR) using ChamQTM Universal SYBR^®^ qPCR Master Mix (Vazyme Biotech Co., Ltd., Nanjing, China) was performed on a LightCycler^®^ 96 platform (Roche, Shanghai, China). The rice *Actin1* gene was used as the endogenous control. All reactions were conducted three times. The 2^-△△Cq^ approach (Livak and Schmittgen, 2001) was used to examine relative expression. The primers used in this work are listed in **Supplementary Table 4**.

### Statistical analyses

For statistical analysis with Student’s *t*-tests, SPSS 26.0 (IBM, USA) was used. To prepare figures, TBtools (Chen et al., 2020) and GraphPad Prism 8.0 software (GraphPad Software, China) were used.

## Results

### Cloning of *Xa47*

**Figure 1:**
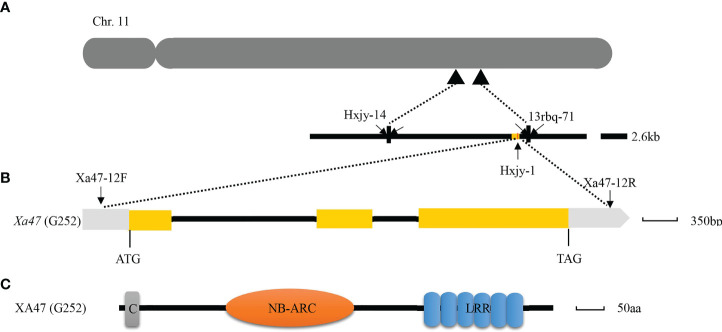
Map-based cloning of *Xa47*. **(A)** Physical mapping of *Xa47* on chromosome 11 of rice based on the Nipponbare reference genome. **(B)** Structure of the *Xa47* gene. Yellow rectangles indicate exons, and the black line indicates introns. **(C)** Prediction of the functional domain of XA47. C, Rx N-terminus; NB-ARC, NB-ARC domain; LRR, leucine-rich repeat protein.

Previously, to isolate *Xa47*, an F2 population obtained by crossing the IL G252 with the susceptible cultivar 02428, which therefore included plants with or without *Xa47*, was inoculated with different strains of Xoo to trigger *Xa47*-mediated BB resistance in rice (Xing et al., 2021). After gene linkage and lesion length analyses, the *Xa47* gene was localized to a 2.6-kb area on chromosome 11 between markers Hxjy-14 and 13rbq-71 (**Figure 1A**). In addition, *Xa47* in G252 was identified as a dominant disease-resistance gene (Xing et al., 2021). According to the reference sequence of the rice cultivar Nipponbare (NPB), the NLR-encoding gene *LOC_Os11g46200* was chosen as the target gene for *Xa47* (Xing et al., 2021).

Homologous cloning was used to clone *Xa47* in sequential rice materials G252 and 02428 (**Supplementary Figures 1A–C**). Notably, the *xa47* sequences in rice materials 02428 and NPB were consistent, whereas the *Xa47* sequences in G252 differed from those sequences (**Supplementary Figure 2**). The gene *Xa47* was 4,240 bp in length, had three exons and two introns (**Figure 1B**), and encoded 802 amino acids (aa) (**Figure 1C**). The protein XA47 (G252) contained a typical NB-ARC domain and a leucine-rich repeat (LRR) protein, consisting of 237 and 178 aa, respectively (**Figure 1C**). In addition, XA47 (G252) also contained an Rx N-terminal domain (**Figure 1C**). Further multisequence alignment and phylogenetic analysis showed that all homologous genes of *Xa47* were distributed in graminaceous crops. Notably, XA47 (G252) was slightly more homologous to EAY84977.1 than to XA47 (NPB) (**Supplementary Figure 3**).

### *Xa47* is in some bacterial blight-resistant rice

**Figure 2:**
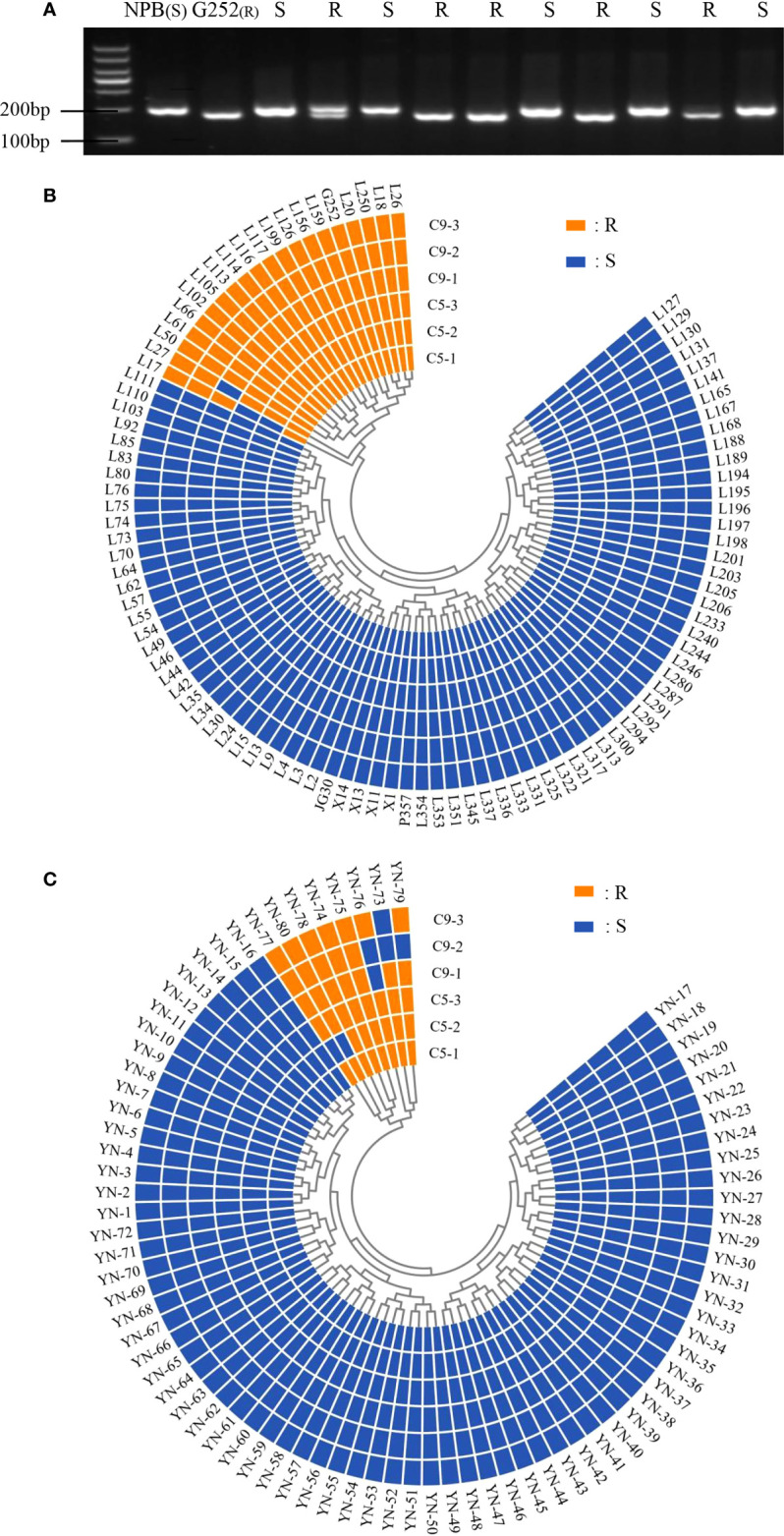
Identification of *Xa47* in 180 rice varieties. **(A)** Detection of rice resistance gene *Xa47* using the molecular marker Hxjy-1. R indicates *Xa47* disease resistance allele fragment of 170 bp; S indicates *Xa47* disease susceptibility allele fragment of 210 bp.NBP was the susceptible control material and G252 was the susceptible control material. **(B)** Reactions of 100 infiltration lines inoculated with C5 and C9 strains of *Xanthomonas oryzae* pv. *oryza* (Xoo). R: resistant (lesion length ≤ 6 cm); S: susceptible (lesion length > 6 cm). **(C)** Reactions of 80 Yunnan (YN) landraces inoculated with C5 and C9 strains of Xoo. R, resistant (lesion length ≤ 6 cm); S, susceptible (lesion length > 6 cm).

The functional marker Hxjy-1 of *Xa47* and homologous cloning were used to detect *Xa47* in 180 rice materials. Twenty of 100 ILs contained *Xa47* (**Figures 2A, B** and **Supplementary Table 2**), and eight of 80 rice landraces contained *Xa47* (**Figure 2C** and **Supplementary Table 3**). To confirm whether the materials were resistant to BB, a disease assessment was performed. Almost all *Xa47*-containing materials showed resistance to C5 and C9 strains (**Figure 2B**).

### *Xa47* is localized in the nucleus and cytoplasm

**Figure 3:**
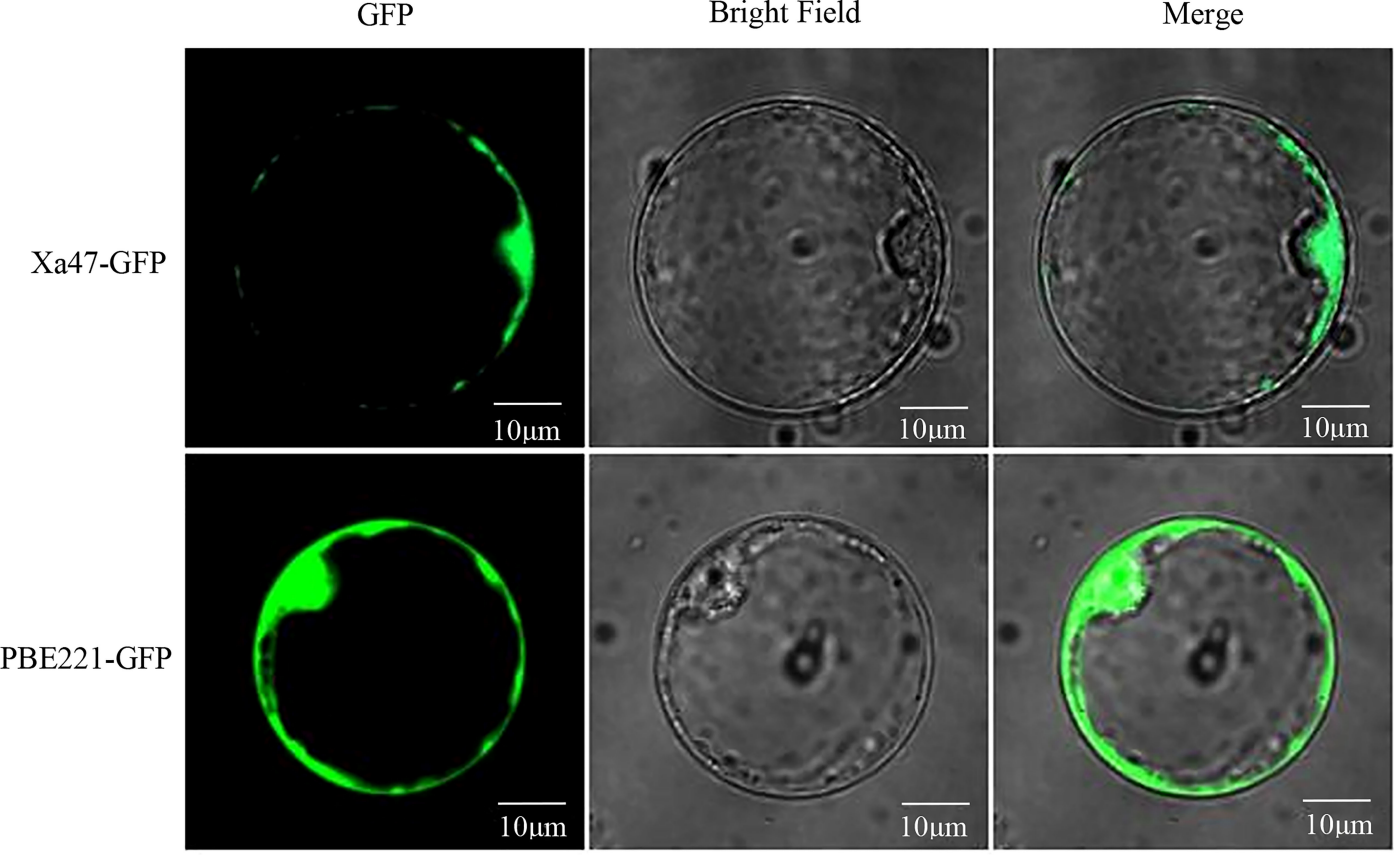
Subcellular localization of XA47 in rice protoplasts. Fluorescence detection of XA47-GFP fusion protein in rice protoplast cells with pBE221::GFP transformed rice protoplast cells as controls.

To determine the subcellular localization of XA47 in cells, a 35S::Xa47-GFP vector was constructed for transient expression in rice protoplasts. Laser scanning confocal microscopy showed that the fluorescence signal of cells transfected with 35S::GFP was uniformly distributed throughout cells, whereas the strong fluorescence signal of cells transfected with 35S::Xa47-GFP was distributed in the nucleus and cytoplasm. Thus, XA47 was localized in the nucleus and cytoplasm (**Figure 3**).

### Loss-of-function mutations of *Xa47* result in loss of resistance against *Xanthomonas oryzae* pv. *oryzae*

**Figure 4:**
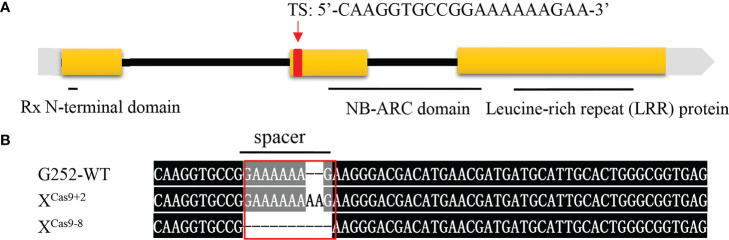
*Xa47* knockout mutant lines. **(A)** Design of target site (TS) sequences. **(B)** Sequence mutations at the *Xa47* target site in two T_0_-generation *X* ^Cas9^ rice plants. The TS sequence is from G252.

Previously, when locating the *Xa47* gene, it was predicted to be a new *R* gene that conferred resistance to Xoo in rice (Xing et al., 2021). Therefore, a sgRNA was designed to specifically target the second exon of *Xa47* (sequence ‘CAAGGTGCCGGAAAAAAGAA’), and it was cloned into a Ubi-Cas9 vector. When G252 was transformed with the constructed knockout vector, 30 T_0_ transgenic plants were obtained. The mutation sites were sequenced, and six mutations were identified in T_0_ transgenic plants. Two homozygous mutant lines were selected, one with two base pairs inserted at the target site (*X* ^Cas9+2^) and one with eight base pairs missing (*X* ^Cas9-8^), which were used in further molecular and phenotypic analyses (**Figures 4A, B**).

**Figure 5:**
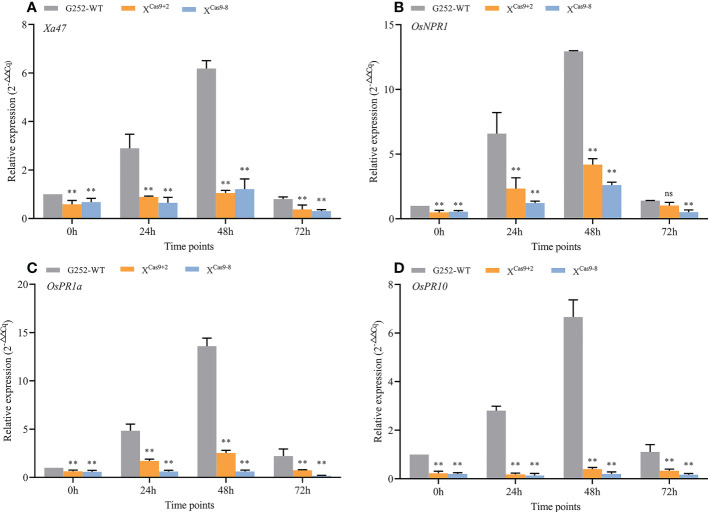
Expression of defense-related genes in *X* ^Cas9^ plants after inoculation with *Xanthomonas oryzae* pv. *oryzae* (Xoo). **(A–D)** Relative expression level of *Xa47*, *OsNPR1*, *OsPR1a*, and *OsPR10* in the G252 and two X^Cas9^ lines at 0, 24, 48, and 72 h post-inoculation with Xoo. Values are the mean ± SD (*n* = 3). Asterisks indicate significant differences compared with the wild type (WT), based on Student’s *t*-tests (**P < 0.01). Each experiment was performed with three biological replicates.

**Figure 6:**
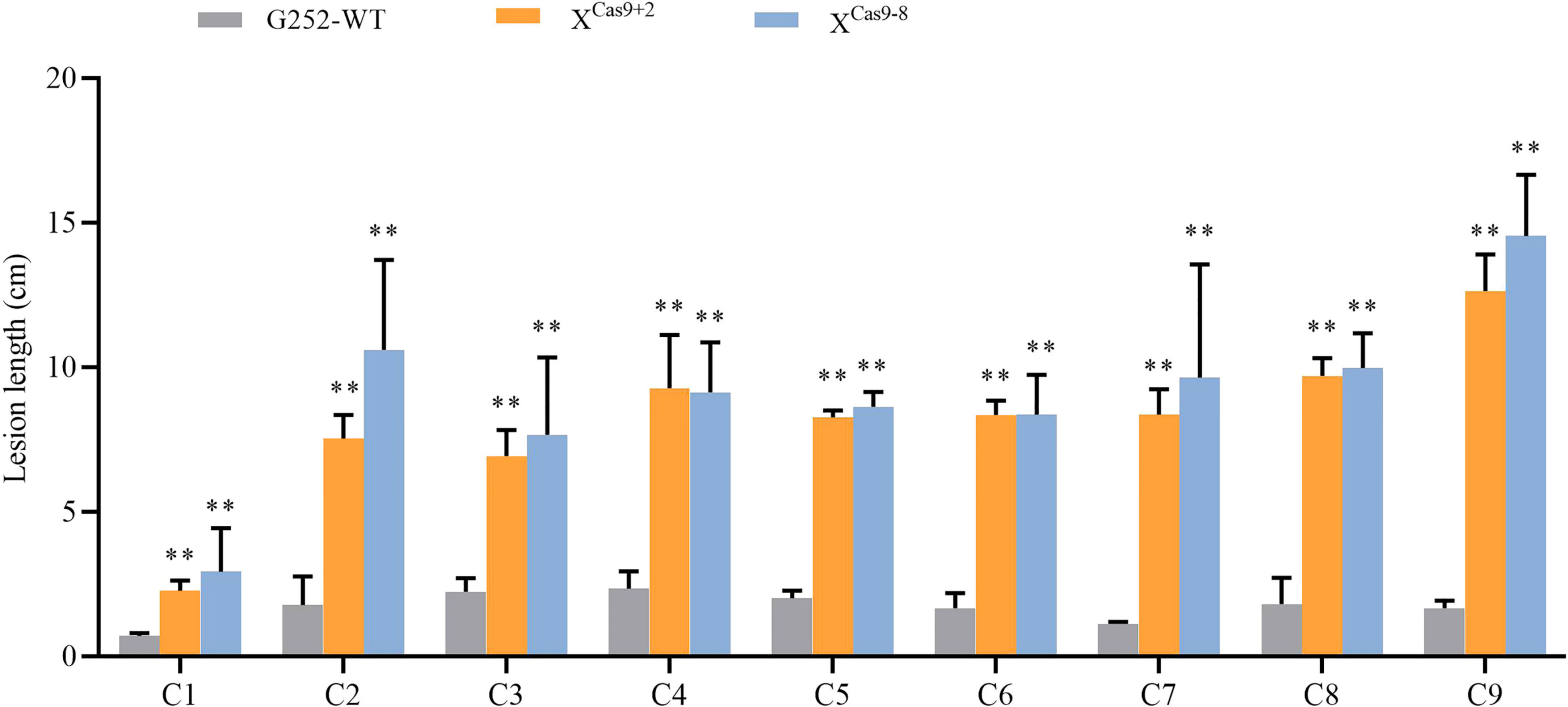
Lesion length of rice after infection with nine *Xanthomonas oryzae* pv. *oryzae* isolates (C1–C9). Values are the mean ± SD (*n* = 9). Asterisks indicate significant differences compared with the wild type (WT), based on Student’s *t*-tests (**P < 0.01). Each experiment was performed with three biological replicates.

The *X* ^Cas9^ and G252 (WT) plants were grown to the late tillering stage, and leaf specimens were collected at 0, 24, 48, and 72 h after inoculation with Xoo and subjected to RT-qPCR. At each time point, the expression level of *Xa47* in *X* ^Cas9^ lines was much lower than that in G252 (WT) lines, which further confirmed the successful knockdown of *Xa47* in G252 (**Figures 5A-D**). Transcript levels of the defense-related marker genes *OsNPR1*, *OsPR1a*, and *OsPR10* were also measured. At nearly all time points, expression levels of the three genes were considerably lower in *X* ^Cas9^ plants than in G252 plants, which suggested that the defense response was affected in mutant plants. Therefore, the two T_1_ *X* ^cas9^ mutants were inoculated with nine Xoo strains at the rice booting stage using the leaf tip-clipping method. At 14 d following inoculation, all *X* ^Cas9^ plants exhibited much longer lesions than those in G252 plants (**Figure 6**). Collectively, the findings revealed that *X* ^Cas9^ mutations caused *Xa47* to lose its resistance to BB.

### Overexpression of *Xa47* increases resistance of JG30 to bacterial blight

**Figure 7:**
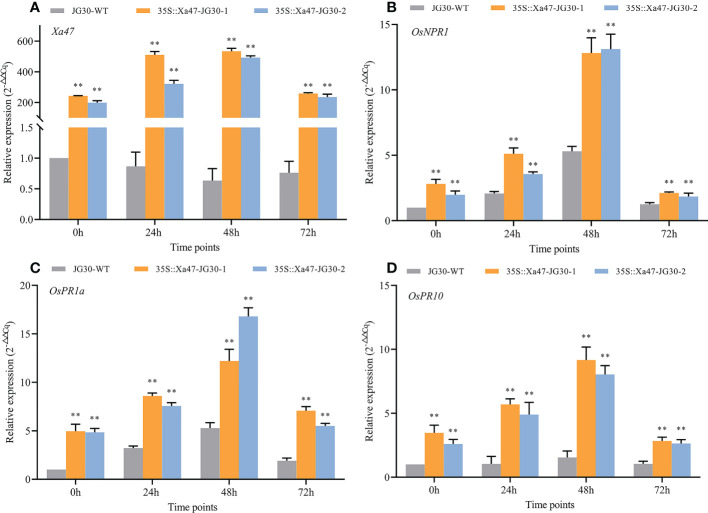
Expression of defense-related genes in 35S::Xa47-JG30 plants after inoculation with *Xanthomonas oryzae* pv. *oryzae* (Xoo). **(A–D)** Relative expression levels of *Xa47*, *OsNPR1*, *OsPR1a*, and *OsPR10* in the G252 and two 35S::Xa47-JG30 lines at 0, 24, 48, and 72 h post-inoculation with Xoo. Values are the mean ± SD (*n* = 3). Asterisks indicate significant differences compared with the wild type (WT), based on Student’s *t*-tests (**P < 0.01). Each experiment was performed with three biological replicates.

**Figure 8:**
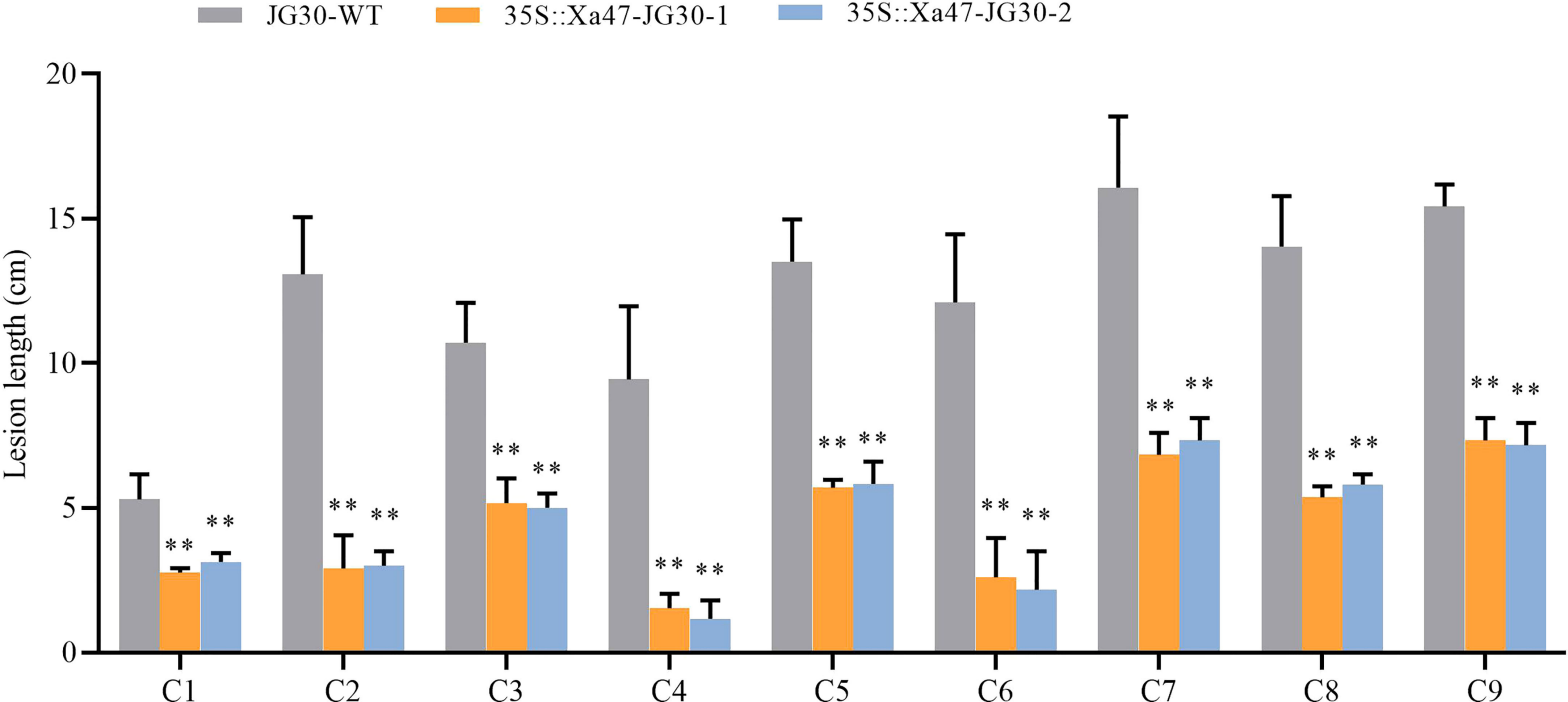
Lesion length of rice after infection with nine *Xanthomonas oryzae* pv. *oryzae* isolates (C1–C9). Values are the mean ± SD (*n* = 9). Asterisks indicate significant differences compared with the wild type (WT), based on Student’s *t*-tests (**P < 0.01). Each experiment was performed with three biological replicates.

The indica rice cultivar JG30 is extremely vulnerable to Xoo because it lacks *R* genes for BB resistance. Notably, the *Xa47* genotype in JG30 was consistent with that of NPB and 02428. To determine whether *Xa47* could increase resistance in JG30 to BB, an expression vector 35S::Xa47 was constructed, and then, *Xa47* was overexpressed in JG30. Overexpression (35S::Xa47-JG30-1 and 35S::Xa47-JG30-2) and JG30 (WT) plants were grown to the late tillering stage, and leaf specimens were collected at 0, 24, 48, and 72 h after inoculation with Xoo and subjected to RT-qPCR. At each time point, the expression level of *Xa47* in 35S::Xa47-JG30 lines was much higher than that in JG30 lines, which confirmed the successful transformation of 35S::Xa47 in JG30 (**Figures 7A-D**). Transcript levels of the defense-related marker genes *OsNPR1*, *OsPR1a*, and *OsPR10* were also measured. At nearly all time points, expression levels of the three genes were significantly up-regulated in 35S::Xa47-JG30 plants compared with JG30 plants. The data indicated that overexpression of *Xa47* in rice could activate its defenses. Therefore, 35S::Xa47-JG30 plants were inoculated with nine Xoo strains at the rice booting stage using the leaf tip-clipping method. At 14 d following inoculation, all overexpression plants exhibited much shorter lesions than those of JG30 plants (**Figure 8**). Notably, the growth of 35S-Xa47-JG30 plants was similar to that of JG30-WT (**Supplementary Figures 4A-H**). Collectively, the findings revealed that overexpression of *Xa47* increased the resistance of JG30 to BB.

### Interfering transcription activator-like effectors reduce resistance mediated by *Xa47*

**Figure 9:**
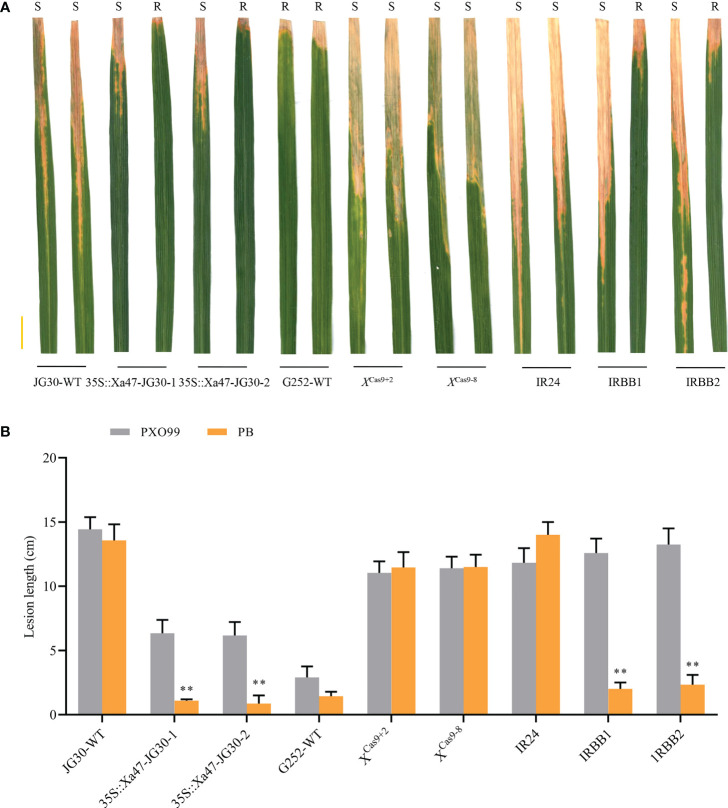
*Xa47*-mediated resistance is attenuated by interfering transcription activator-like effectors. **(A)** Photographs of infected leaves of different rice materials 2 weeks after inoculation with *Xanthomonas oryzae* pv. *oryzae* strains PXO99 and PB. Scale bar: 2 cm. Two leaves of each rice variety show disease spots after inoculation with PXO99 and PB. **(B)** Lesion lengths on infected leaves in **(A)**. Values are the mean ± SD (*n* = 9). Asterisks indicate significant differences compared with the wild type (WT), based on Student’s *t*-tests (**P < 0.01). Each experiment was performed with three biological replicates.

As NLR genes specific to rice resistance to BB, *Xa1* and its alleles (*Xa2*, *Xa14*, *Xa31*, and *Xa45*) have identified various TALEs (PthXo1 and Tal4 and Tal9d) to induce resistance to Xoo. Nonetheless, certain iTALEs, such Tal3a and Tal3b in PXO99, reduce this resistance (Zhang et al., 2020). Notably, *Xa47*, a newly identified NLR gene in rice BB resistance after *Xa1* and its alleles, also possessed a functional structural domain similar to that of *Xa1*. Nevertheless, XA47 had little sequence similarity to XA1 and XA2/XA31 and XA14 and XA45 proteins. To investigate whether *Xa47* was influenced by iTALEs, PXO99 and PB (which does not secrete iTALEs) strains were used for identification. After inoculation with PXO99, JG30, 35S::Xa47-1 and 35S::Xa47-2, and *X* ^Cas9+2^ and *X* ^Cas9-8^ plants were as susceptible as IRBB1, IRBB2, and IR24 plants, but the opposite was observed for G252 (**Figures 9A, B**). By contrast, the resistance of 35S::Xa47-1 and 35S::Xa47-2 and G252 plants after PB inoculation was similar to that observed in IRBB1 and IRBB2 plants (**Figures 9A, B**). However, *X* ^Cas9+2^ and *X* ^Cas9-8^ and JG30 plants after PB inoculation were as susceptible as IR24 plants (**Figures 9A, B**). The findings suggested that iTALEs can also reduce *Xa47*-mediated resistance.

## Discussion

The *R* genes have essential roles in the competition between rice and Xoo, and the discovery of new *R* genes will be crucial to rice success. Nearly one-third of the *Xa* genes are localized on chr. 11, including *Xa3/Xa26*, *Xa4*, *Xa10*, *Xa21*, *Xa22*, *Xa23*, *Xa30*, *Xa32*, *Xa35*, *Xa36*, *Xa39*, *Xa40*, *xa41*, *Xa43*, *xa44-1*, *xa44-2*, *Xa46*, and *Xa47* (Sun et al., 2004; Xiang et al., 2006; Tian et al., 2014; Wang et al., 2014; Kim et al., 2015; Zhang et al., 2015; Kim, 2018; Chen et al., 2020; Neelam et al., 2020; Xing et al., 2021). Even though many *R* genes have been identified, relatively few have been cloned or used in breeding. For example, *Xa4*, *Xa21*, and *Xa23* have been used most frequently in breeding for disease resistance in hybrid rice (Balachiranjeevi et al., 2018). The primary cause of this problem is that most *R* genes have disadvantages, such as a limited resistance spectrum or insufficient resistance. To overcome such problems, the exploration for new *Xa* genes with broad-spectrum resistance must be continuous. In addition to identifying new *Xa* genes with broad-spectrum resistance, multiple *Xa* genes can be aggregated into the same variety to produce disease-resistant varieties (Dash et al., 2016; Wu et al., 2019). Additionally, rice variants with broad-spectrum resistance can be developed using gene editing technologies (Tao et al., 2021).

In this study, a new NLR gene, *Xa47*, was cloned from G252 (**Figure 1C**). The cloning of *Xa47* increased the number of NLR genes for BB resistance to two (*Xa1* and *Xa47*, not including alleles). Allelic variety in functional genes is prevalent in plants, and that variation is essential for plant evolution (Zhang et al., 2020). In rice, *Xa1* and *Xa2/Xa31*, *Xa14*, and *Xa45* are alleles of one another, and there are sequence differences among them. Notably, *Xa47* and *xa47* are alleles of one another, and there were sequence differences between them (**Supplementary Figure 2**). This result also indicated that *Xa47* is variable at natural loci, which warrants additional study. Most plant *R* genes code for NLRs, which are composed of an N-terminal signaling domain, a central nucleotide-binding region (NBS), and a C-terminal leucine zipper repeat region (LRR) (Mermigka et al., 2020). The NLRs are split into two types based on the N-terminal structure. The TIR-NBS-LRR has a toll/interleukin receptor (TIR) structural domain, whereas the CC-NBS-LRR contains a coiled-coil (CC) motif (van Wersch et al., 2020). In addition, some NLRs contain other structural domains, such as kinase, WRKY, and BED finger domains (Le Roux et al., 2015; Kroj et al., 2016; Bailey et al., 2018). In this study, XA47 was a typical CC-NBS-LRR resistance protein (**Figure 1C**), whereas XA1 was not a typical CC-NBS-LRR resistance protein and contained other structural domains (Zhang et al., 2020). The results indicated that XA47 and XA1 have distinct functions in disease resistance.

The NLR genes are critical in plant immune systems, because they recognize specific pathogens and activate resistance responses (Lei and Li, 2020). The NLR gene-mediated disease resistance response has a dose effect (Howles et al., 2005). Simultaneously, NLR proteins can lead to activation of plant disease resistance responses (Tao et al., 2000). However, during normal plant development, NLR genes are strictly regulated to prevent induced self-activating reactions (Du et al., 2021), hence preventing abnormal plant growth and development. In this study, in the mutant lines overexpressing *Xa47*, cell death was not observed (**Supplementary Figures 4A-H**), and thus, the overexpression of *Xa47* did not spontaneously activate the rice ETI response to the HR-like phenotype.

The gene *OsNPR1* is essential for plant disease resistance, especially for resistance to rice bacterial blight (Chern et al., 2005). The *OsNPR1* gene was expressed in response to Xoo at different times after inoculation in 35S::Xa47-JG30 plants, and *OsNPR1* expression was significantly higher in 35S::Xa47-JG30 plants than in those of the wild type. Expression of the *OsNPR1* gene in *X* ^Cas9^ plants was also induced by Xoo at different times after inoculation, but expression was significantly lower in *X* ^Cas9^ plants than in those of the wild type. Therefore, *Xa47* can strongly activate the expression of *OsNPR1* and thus stimulate OsNPR1-mediated disease resistance response in rice. Expression of pathogenesis related(*PR*) genes tends to promote plant cell death, hence inhibiting the spread of harmful microorganisms (Kim et al., 2011; Hu et al., 2017). Notably, both disease process-related genes *OsPR1a* and *OsPR10* were significantly activated with significantly higher expression in the 35S::Xa47-JG30 strain than in the wild type when resisting infestation by Xoo (**Figures 7A-D**). Both *OsPR1a* and *OsPR10* were also significantly activated with significantly higher expression in G252 than in *X^Cas9^* plants in response to Xoo infestation (**Figures 5A-D**). The results suggested that *Xa47* regulates the resistance response in rice.

Identifying the level of resistance of an *R* gene to pathogens is also an assessment of the value and potential of the application of that gene (Feng et al., 2022). In this study, *Xa47* was determined to be an *R* gene with broad-spectrum resistance by multiple methods. There were three essential observations: (1) *Xa47* occurred in some BB-resistant rice; (2) loss-of-function mutations of *Xa47* led to loss of resistance against Xoo; and (3) overexpression of *Xa47* increased the resistance of JG30 to BB (**Figures 2**-**7**). China is a rice-producing nation, and most rice-growing regions are affected by Xoo (Lu et al., 2021). Representative Xoo strains, including C1, C2, C3, C4, C5, C6, C7, C8, and C9, have been discovered in the disease-endemic regions. Therefore, the use of isolates from diverse rice-growing regions in China to evaluate the resistance of rice lines to BB, particularly during gestation, was an important aspect of this work.

Previously, it was reported that iTALEs reduce *Xa1*-mediated resistance (Zhang et al., 2020). In this study, the resistance mediated by *Xa47* was also inhibited by iTALEs. Notably, G252 showed resistance to PXO99 and PB strains. In 35S::Xa47-JG30 plants inoculated with PXO99 strains, lesion length was approximately 6.0 cm, whereas the lesion length in JG30 and IRBB1 plants was more than 10 cm (**Figures 9A, B**). The difference in resistance between G252 and 35S::Xa47-JG30 plants might be attributed to differences in genetic background. Moreover, the results indicated that *Xa47*-mediated resistance exceeds *Xa1* resistance. The isolation and identification of *Xa47* will not only reduce yield loss of rice due to Xoo infection but will also enable crop breeders to include *Xa47* in breeding programs. Notably, 100 BC_2_F_16_ generations of Yuanjiang common wild rice IL materials, which possess a diverse antimicrobial spectrum, were available for selection of rice varieties. The varieties containing *Xa47* (YN-80, YN-79, YN-78, YN-77, YN-76, YN-75, YN-74, and YN-73) that were isolated from 80 local rice varieties can be used as novel donors in rice breeding efforts. Therefore, the results of this study can help expedite the development of leaf blight-resistant cultivars in Yunnan Province and adjacent areas.

## Data availability statement

The original contributions presented in the study are included in the article/**Supplementary Material**. Further inquiries can be directed to the corresponding authors.

## Author contributions

ZC and LC designed the research. YL, XK, YZ, FY, DZ, CJ, LL, JL, TY and LW performed the experiments and analyzed the data. YL and LC wrote the manuscript. QZ, SX and BW revised the manuscript. All authors contributed to the article and approved the submitted version.

## Funding

This study was supported by the Yunnan Yanlongan Academician Expert Workstation (no. 202005AF150032), the Yunnan Seed Seed Industry Joint Laboratory (no. 202205AR070001), the Yunnan Basic Research Special Project (no. 202001AT070015 and 202001AT070003), the Yunnan Science and Technology Talents and Platform Project (no. 2019HB034 and 202005AM070029), the Yunnan Young Top Talents Special Project (no. YNWR-QNBJ-2018-284), the National Natural Science Foundation Regional Project (no. 31960374), and the Central Guidance Local Science and Technology Development Fund Plan (no. 202207AB110012), respectively.

## Acknowledgments

We are grateful for the Xoo strains that were provided by Dr. Gongyou Chen (College of Agriculture and Biology, Shanghai Jiaotong University, Shanghai, China).

## Conflict of interest

The authors declare that the research was conducted in the absence of any commercial or financial relationships that could be construed as a potential conflict of interest.

## Publisher’s note

All claims expressed in this article are solely those of the authors and do not necessarily represent those of their affiliated organizations, or those of the publisher, the editors and the reviewers. Any product that may be evaluated in this article, or claim that may be made by its manufacturer, is not guaranteed or endorsed by the publisher.
