# Supplementary material for: A new NLR disease resistance gene Xa47 confers durable and broad-spectrum resistance to bacterial blight in rice
Source: Front Plant Sci. 2022 Nov 24;13:1037901. doi: 10.3389/fpls.2022.1037901 (PMC9730417; doi:10.3389/fpls.2022.1037901)
Supplement: Supplementary file 1 [file DataSheet_1.docx]

Supplementary Material

# Nucleotide sequence of the *Xa47* gene

# >*Xa47*(G252)

# ATGACGGGGGAGGAAGTCGATGCTTTGTGCAAGGATGAGTTGATGGCGGAGGTGCGTGAGCTGTCCTACGACATGGACGACGCCATCGACGAATTCTTCTTAGAGGAGCCCATGGCGGGCGGCGACGGTGGCCCTTTCGATGAGCTCAAGACAAGAGTTGAGGATGTCTCCAAGCGGTTCTCCGACAGCCGGCGGTGGAGGCCACAGGTGGAGCAACATCAACCATCCCTAACCGCCGCAACCGTAGACTGTCCACCTCCTCACGCTCGCTTCGTCCACAACATGATGGATGTGTCAGAGCTCGTGGAGATGGACAAACTACATGAGACAGAGCTCATCAAATTGCTGGAACAAGGTGCGGACACAAGCATATATGCTTCCCGGTGGCGCATCGCAACACCATGGCATGATAAGGAGGTAAAGACGACATCCTTTTATTTCTTTTTTATCTCTACTTCTCTATTTATATATTATATTATAAAAATTTAAAATGTTTTCGCTGTGTGTATTTTGGTACGTCGTGGCTATCTTCGTATCGTATTCGATCTCCCGTTCAGTATGTTTTGTGTTGTACGTCTCTAGCTTCCAGATATATCATATATCTTTCCATTCCTATGTTATTCTTTCTTTCCAAATTCCAATTATTAATTATACCTCATTTAATGAGGGACATTTATACCATTTTATTTCAGTAGTAATTTCATCCTTTCTCCTAGTGCTAGAAGTGCACTTGCATGAGGATGGAGGCAAAGCTGAAATACCCATTCGTTGTGATGAAATTTTAAAAGCGAACTCTACCGTAAACTCTAAATTAAGCCCCAAAAATTATAATGCAAGTATTCCTGTTTTAATCAATATTTGGCATCATTTTTTTTACAATGTATAATTCAGCTACACAATTTTCTCATTTTTTTTTACAATTGCTTATAATTAAGCTACACAAGCTACCAATTCAGCTACACAATTGGTACATACTACTATACTAGCCAAATACCCGTGATTTGCTACGTATTAAAACAAATTAATAGTGAGATTTTTGGGGCAATTAATTTGGTTTTGTAGAAGTTATATCATAGAGAAATTATTTTATAACGGTAAAGTTGGTTGAAAATATGATGGATAATGTGGTAAATAAAAAAAGTACTATAGTTGGTGGCAGATTCACTGCCATTGCCCTCTTTTGAAAGGAATATAGAATTTTATCTTGTAGAAGTTATATTGTACAAGTGAAATATGATGGAATCATATATGTAGAATAAAACATTAAAGTATGTGGGGGTATTTGGTTGAAAATATGATGGAGTATGTGGTAAATAGAAAAAAAATACTATACTTGATGATGGGGCGATGATAGATTCACTGCCACCACCATTGCATTTTTTTTAAAAGGAGTATATAAACATATATAGGAACCCATCTACTCATTACTTGGTAAGAGGTCTTACTTGGTAATTGTGCTGGACGGTAGCATGCCAGTTTACCATTCATCATCATTATTGGATTCCTTTTTTTTCTTAAAAAAAGGTATAATATGATATGCAACTTCTTAATTGCTTTATTTCTTTTCTAGATTAATTTTAGATAAAAATTTTATTGGATATGGATCAGCTAGCGTAGTAAAAAGTGAACGATACATGAGAAAAAAGATTGATTTGACAAAACAAAAACACAACCCATTAAATTGGAGCGTCTTATTCCCGTAGACTGTAACAGAATGGTTCGGTGATATGATCAACTAATGTTTTTTGTTGCAGCAAAGTATTGTGGTCAAGGTGCCGGAAAAAAGAAGGGACGACATGAACGATGATGCATTGCACTGGGCGGTGAGTTCGTTGCATGGAGTGCCCTCGGGTGGTACGTCTGGAGATTGTAGTAGGTTGCAGTTGGATGGTGAAGGCGCGAACATCCGCAAGCTCTTGTCCACCCTCCGGAATAAGGTGGGCCACGCCCAGTTGGTGCAGGTCGAGGATAAGAGAAAAAGGGTAGAGGAGGCGACGAAGCCTTGTGAATTTCACGAGGTCAAAACAATATGCATCCTTGGATTGCCTGGCGCAGGCAAAACAACTCTTGCAAAACTGTTGTACTCCCATCACTCAACGACAGAGCAGCAATTCCAACACCGGGCTTTCGTGTCACTCTCTCCGGGTGCCAATCTCACCGACACTCTTACTGATATTTTATTGCAAGTAGGAGCATATAATGATGATGCAACACCATATTGTGGGACCGGAACACCGCACCAACAGTATCTCATTGACAACATATCAGCTTATCTCATTGGCAAAAAGTAAGCAGAGTTCTTTAGAATGATGTTATTTTAAATAATATTATTTTTTTTAAAAAAAAATTAACAATGTGTATTTGATGGAATTAATAAAAATATGTTTTAAGAGAAATTAATAAAAAATATTTGATATCAAATTCTGCAGGATGGTCTTAATTAGAATTTCTATAAAAAGAGAGTAGATGAGAAACACCCAGGGGCTCTTCTGGCTAGCTCCACAAGCCAACCTATTATGTTTGAAGCCTCACCCCTACCTATTTAATATTAGGTCTTTCTCTAATATTCGCTATTTATTTGATATTAAATCCTTCCCTAATATTCGTGTTTTTAAAAGAGAGTAGATGACAAACAGACATCAAATTAAGCTGATTGTTTTTCGATCATCTCAAAGGGGAAGCTTCTCATGTGGGTGGACTCATATCTTCGAAATTATTATATAGTTGCATGTATTAGTGCTAATATATTGAGGCTTATTTACTTTTTTTCAACTTCTAAAGATATCTTATTATAATTGATGACGTTTGGCGCTGGGAAGAGTGGGAAGTCATCAGAAAGTCCATTCCAAAGAATGATCTGGGTAGCAGAATAATCATGACTACTCGTCTTAATTCAATAGCTGAGAAGTGTCGCAATGATGACATGGATGCGTTTGTTTATGAAACTGAGGCTCTGGATTATGTGGATGCTTGGCTGTTGTGTGACAAGGTAGCAAGAAAGTCTGTCACATGTATGAACATTAATCCATGCTATGATATCGTGGACGTGTGCTATGGTATGCCGTTAGCACTAATTCGTGTGTCGTCAGCATTGGCAGAAGAGATACAAGCTTTAGACAGTGATGAATGGCAAATATGGAGGGCTCTGAGACGGGTAGAGGATGGTATTTTGGACATCCCATCCTTGAAGCCATTGGCAGAGAGTTTATGCCTTGGTTACGACCATCTTCCTCTCTATCTGAGGACTTTGTTGTTATGTTGTAGTGTGTACCATTGGCTTGATGGTGGGATTGTTCAAAGGGGCCGTTTGGTCACAAGGTGGATTGCTGAAGGATTTGTTTCAGAAGAGAAAGCAGCAGAAGGTTACTTTGATGAGCTTGTCGACAGAGGATGGATTAAGCATAGAGGGTGGAACGAGTATGAGATCTACCCTATGATGCTGGCCATCCTTAGATACAAGTCGAAGGAGTACAATTTTGTAACTTGTTTGGGTACGGGATTTGATACTTGTACTAGTGCATCTCTATCCTACTCCTCTCCAACAATGGCGATTCGCCGGCTTTGTCTTCAAAGGGGGTACCCAATGAAATGCTTCTCAAGTATGGATGTGTCACACACTCGCAGCCTTGTTATCCTTGGCGACGTGATAGGAGTCCCCTTGGATATGTTTAAAAGATTGCGAGTGTTGGACCTTGAAGATAATATCGGTATAGAGGACTCCCACCTGAAGAAGATATGTGAGCAGCTAGAGAGCCTCAGGCTGCTCAAGTACCTAGGTCTCAAGGGTACGCGAATCACTAAGCTCCCACAGGAGATACAGAAGCTGAAGCATCTGGAGATTTTGTACGTGAGGAGCACAGGCATCAAAGAGCTCCCACGGGAGATCGGGGAAGTGAAACAACTGCGGACTCTGGACGTGAGGAACACGCGGATCAGCGAGCTCCCGTCGCAGATCGGGGAGCTCAAACATCTGCGGACTCTGGACGTGAGGAACACGCGGATCAGCGAGCTCCTGTCGCAGATCGGGGAGCTCAAACATCTGCGGACTCTGGACGTGAGGAACACGCGGATCAGCGAGCTCCCGTCGCAGATCGGGGAGCTCAAACATCTGCGGACTCTGGACGTGAGGAACACGCGGACTTCTATATTTTTTTATTCTAGAAGAAGAATAAAAAAATATAGAAGTACTGATATCTGGCTCTCTGCACGTGACATGCATCATACATGTATATGGTATTAA

> *xa47* (NPB, 02428, JG30)

ATGGCCGTATACAGCGTCGCCACGGGGGCCTTGGCTCCCGTCCTATCGAAGCTCTCCGCTTTGCTGGGCGACGAGCACTTGGATCTTGCGGAGAGGACCCGGAGCGACGCCATGTTCATCAGGTCCCAGCTGGAGGCCGTGCACTCTCTCCTCCTCCCGAGGATTAGTTGGGGAATGACGGGGGAGGAAGTCGATGCTTTGTGCAAGGATGAGTTGATGGCGGAGGTGCGTGAGCTGTCCTACGACATGGACGACGCCATCGACGAATTCTTCTTAGAGGAGCCCATGGCGGGCGGCGACGGTGGCCCTTTCGATGAGCTCAAGACAAGAGTTGAGGATGTCTCCAAGCGGTTCTCCGACAGCCGGCGGTGGAGGCCACCGGTGGAGCAACATCAACCATCCCTAACCGCCGCAACCGTAGACTGTCCACCTCCTCACGCTCGCTTCGTCCACAACATGATGGATGTGTCAGAGCTCGTGGAGATGGACAAACAACATGAGAAAGAGCTCATCAAATTGCTGGAACAAGGTGCGGACACAAGCATATATGCTTCCCGGTGGCGCATCGCAACACCATGGCATGATAAGGAGGTAAAGACGACATCCTTTTATTTCTTTTTTATCTCTACTTCTCTATTTATATATTATATTATATTATATTATAAAAATTTAAAATGTTTTCGCTGTGTGTATTTTGGTACGTCGTGGCTATCTTCGTATCGTATTCGATCTCCCGTTCAGTATATTTTGTGTTGTACGTCTCTAGCTTCCAGATATATCATATATCTTTCCATTCCCATGTTATTCTTTCTTTCCAAATTCCAATTATTAATTATACCTCATTTAATGAGGGACATTTATACCATTTTATTTCAGTAGTAATTTCATCCTTTCCTAGTGCTAGAAGTGCACTTGCATGAGGATGGAGGCAAAGCTGAAATACCCATTCGTTGTGATGAAATTTTAAAAGCGAACCCTACCGTAAACTCTAAATTAAGCCCCAAAAATTATAATGCAAGTATTCCTGTTTTAATCAATATTTGGCATCATTTTTTTACAATGTATAATTCAGCTACACAATTTGCTCATTTTTTTTTACAATTGCTTATAATTAAGCTACACAAGCTACCAATTCAGCTACACAATTGGCACATACTACTATACTAGCCAAATACCCGTTATTTTCTACGTATTAAAACAAATTAATAGTGAGATTTTTGGGGCAATTAATTTGGTTTTGTAGAAGTTATATCATAGAGAAATTATTTTATAACGGTAAAGTTGGTTGAAAATATGATGGAGAATGTGGTAAATAAAAAAAATACTATAGTTGGTGGCAGATTCACTGCCACCGCCATTGCCCTCTTTTGAAAGGAATATAGAATTTTATCTTGTAGAAGTTATATTGTACAAGTGAAATATGATGGAATCATATATGTAGAATAAAACATTAAAGTATGTGGGGGTATTTGGTTGAAAATATGATGGAGTATGTGGTAAATAGAAAAAAAATACTATACTTGATGATGGGGCGATGATAGATTCACTGCCACCACCATTGCATTTTTTTAAAAGGAGTATATAAACATATATAGGAACCCACCTACTCATTACTTGGTAAGAGGTCTTACTTGGTAATTGTGCTGGACGGTAGTATGCCAGTTTACCATTCATCATCATTATTGGATTCCTTTTTTTTCTTAAAAAAAGGTATAATATGATATGCAACTTCTTAATTGCTTTATTTCTTTTCTAGATTAATTTTAGATAAAAATTTTATTGGATATGGATCAGCTAGCGTAGTAAAAAGTGAACGATACATGAGAAAAAAGATTGATTTGACAAAACAAAAACACAACCCATTAAATTGGAGTGTCTTATTCCCGTAGACTGCAGTAACAGAATGGTTCGATGATCAACTAATGTTTTTTGCTGCAGCAAAGTACTGTGGTCAAGGTGCCGGAAAGAGAGTGGGGCTTCCCGGACAATCGGAACAGTCCATTTATATGGGCGAGTGATTCGTTTGAACGATTGCGTTCGGGAAGTTTGTGTGGAGATACGTTGCGGTTGGATGGTGAAGGCGCGAACATCCGCAAGCTCTTGTCCACCCTCCGGAATAAGGTGGGCCGCGCCCAGTTGGTGCAGGTCGAGGATAAGAGAAAAAGGGTAGAGGAGGCGACGAAGCCTTGTGAATTTCACGAGGTCAAAACAATATGCATCCTTGGATTGCCTGGCGCAGGCAAAACAACTCTTGCAAAACTGTTGTACTCCCATCACTCAACGACAGAGCAGCAATTCCAACACCGGGCTTTCGTGTCACTCTCTCCGGGTGCCAATCTCACCGACACTCTTACTGATATTTTATTGCAAGTAGGAGCATATAATGATGATGCAACACCATATTGTGGGACCGGAACACCGCACCAACAGTATCTCATTGACAATATATCAGCTTATCTCATTGGCAAAAAGTAAGCAGAGTTCTTTAGAATGATGTTATTTTAAATAATAATATTTTTTTTTAAAAAAAAATTAACAAACATGTTGATGTGTATTTGATGGAATTAATAAAAATATGTTTTAAGAGAAATTAATAAAAAATATTTGATATCAAATTCTGCAGGATGGTCTTAATTAGAATTTCTATAAAAAGAGAGTAGATGAGAAATACCCAGGGGTTCTTCTGGCTAGCTCCACAAGCCAATCTATGTTTGAAGCCTCACCCCTACCTATTTATTTAATATTAGGTCTTTCCCTAATATTCGCTATTTATTTGATATTAAATCCTTCCCTAATATTCGTGTTTTTAAAAGAGAGTAGATGACAAACAGACATCAAATTAAGCTGATTGTTTTTCGATCATCTCAAAGGGGAAGCTTCTCATGTGGGTGGACTCATATCTTCGAAATTATTATATAGTTGCATGTATTAGTGCTAATATATTGAGGCTTATTTACTTTTTTTCAACTTCTGAAGGTATCTTATTATAATTGATGACGTTTGGCACTGGGAAGAGTGGGAAGTCATCAGAAAGTCCATTCCAAAGAATGATCTGGGTAGCAGAATAATCATGACTACTCGTCTTAATTCAATAGCTGAGAAGTGTCGCAATGATGACATGGATGCGTTTGTTTATGAAACTGAGGCTCTGGATTATGTGGATGCTTGGTTGTTGTGTGACAAGGTAGCAAGAAAGTCTGTCACATGTATGAACATTAATCCATGCTATGATATCGTGGACATGTGCTATGGTATGCCGTTAGCACTAATTCGTGTGTCGTCAGCATTGGCAGAAGAGATACAAGCTTTAGACAGTGATGAACGGCAAATATGGAGGGCTCTGAGACGGGTAGAGGATGGTATTTTGGACATCCCATCCTTGAAGCCATTGGCAGAGAGTTTATGCCTTGGTTACGACCATCTTCCTCTCTATCTGAGGACTTTGTTGTTATGTTGTAGTGTGTACCATTGGCTTGATGGTGGGATTGTTCAAAGGGGCCGTTTGGTCACAAGGTGGATTGCTGAAGGATTTGTTTCAGAAGAGAAAGCAGCAGAAGGTTACTTTGATGAGCTTGTCGGCAGAGGATGGATGAAGCATAGAGGGTTGAACGAGTATGAGATCCACCCTATGATGCTGGCCATCCTTAGATACAAATCGAAGGAGTACAATTTTGTAACTTGTTTGGGTACGGGATCTGATACTTGTACTAGTGCATCTCTATCCTACTCCTCTCCAACAATGGCGATTCGCCGGCTTTGTCTTCAAAGGGGGTACCCAATGAAATGCTTCTCAAGTATGGATGTGTCACACACTCGCAGCCTTGTCATCCTTGGCGACGTGATAGGAGTCCCCTTGGATATGTTTAAAAGATTGCGAGTGTTGGACCTTGAAGATAATATCGGTATAGAGGACTCCCACCTGAAGAAGATATGTGAGCAGCTAGAGAGCCTCAGGCTGCTCAAGTACCTAGGTCTCAAGGGTACGCGAATCACTAAGCTCCCACAGGAGATACAGAAGCTGAAGCAACTGGAGATTTTGTACGTGAGGAGCACAGGCATCGAAGAGCTCCCATGGGAGATCGGGGAATTGAAACAACTGCGGACTCTGGACGTGAGGAACACGCGGATCAGCGAGCTCCCGTCGCAGATCGGGGAGCTCAAACATCTGCGGACTCTGGACGTGAGTAACATGTGGAATATCAGCGAGCTGCCGTCGCAAATCGGGGAGCTGAAGCATCTACAAACTCTGGATGTGAGGAACACGTCAGTGAGAGAGCTGCCATCGCAAATCGGGGAGCTGAAGCATCTGCGGACTCTGGATGTGAGGAACACGGGGGTGAGAGAGCTGCCATGGCAAGCTGGCCAGATCTCGGGATCGCTGCACGTGCATACAGATGACAGTGACGAGGGCATGCGGCTGCCAGAAGGCGTATGCGAAGATCTGATCAAGGGTATTCCCAAGGCTGAGCTCGCAAAGTGCAGTGAGGTCCTATCCATCAATATTGTCGATCGTTTAGGATCTCCCCCTATTGGCATATTCAAGGTTATTGGCTTGCACAAGAGTATCCCGAAGCTGATCAAAGATCATTTCAATGTTCTTTCTTCCCTAGACATCAGGCGGTACAACAAGCTAGAGGAGGATGACCATGAGTTTCTAGCCAACAATATGCCTAACCTCCAGATGCTTGTACTGAGGTTCGAGGCCCCACAAAGAGAGCCCATCATCATTAACCGCACAGGCTTCCAGATGCTGGAGAGATTCCTTGTGGAGAGCCGGGTGCCACGGATAACCTTCCAGGAAGGAGCCATGCCCAAGCTCAAGCATCTCGAGTTTAAGTTCTACGCTGGCCCACCAAGCAAAGATCCCATAGGAATCACCCACCTCAAGAGCCTCCAAAAGGTGGTCTTTCGCTGCTCCAAATGGTACAAGAGCGACAACCCTGGCATCAAGGCTGCCATTGACGTCGTGAAGAAAGAAGCAAGGCAGCATCCCAACCGGCCGATCAGCCTTCTCATCACTGAGGGCGATAAGGAGGTACCGAATATTGAGGCACACGGGAGCAGTGAAAACATTGTCGTTGTCCACGCTGCTCCTGACGACGCCATCAGTTGCTCTAGCTGCGGCCGAACCAGCACTAGTATCCAAGAGGGAACAGTCCGAGATCGAATACCAGCTATGGATTTGTTCTGGCCGGAGTTTAACAGCTATGAAAAAGCAAAAAGAAACTAG

# Note: The nucleotide sequences with gray background are exon regions and the rest are intron regions.

# Supplementary Figures and Tables

## Supplementary Figures

##
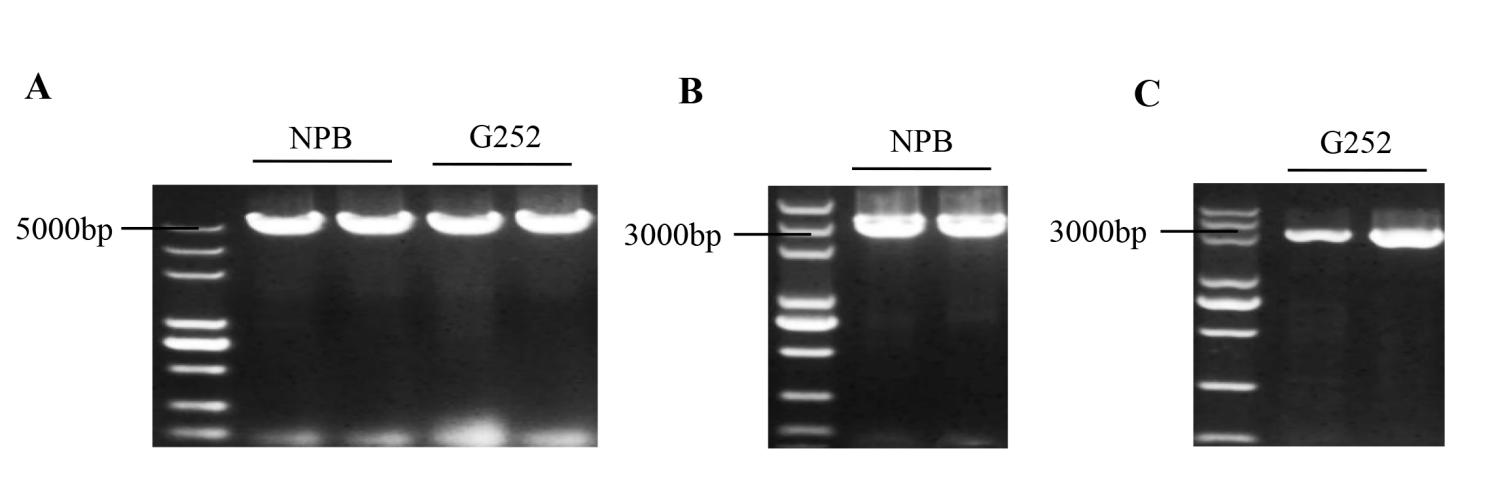


Figure 1 | Cloning of *Xa47* gene. (**A-C**)Target segments of the *Xa47* and *xa47* genes' full-length and CDS sections were amplified by PCR as follows: *Xa47* (G252) and *xa47*(NPB) (**A**), *xa47*-CDS (**B**), and *Xa47*-CDS (**C**).

Figure 2 | Comparison of the *Xa47*(G252) and *xa47*(NPB) CDS sequences. The orange and blue colors indicate identical and different base sequences, respectively.

Figure 3 | Phylogenetic tree of XA47 homologous protein.


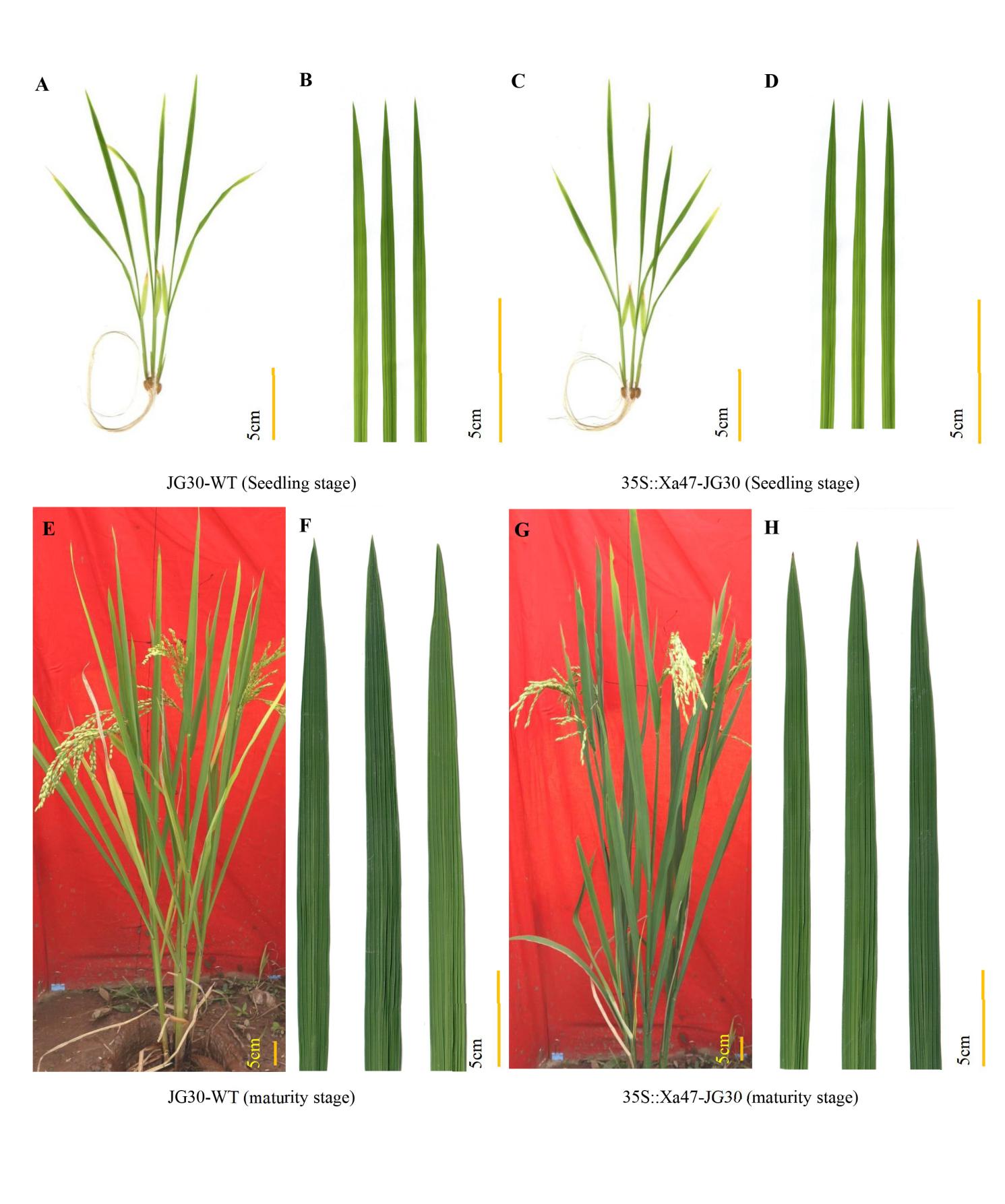


FIGURE 4 | Overexpression of *Xa47* has no effect on the rice phenotype. (**A-D**) JG30-WT and homozygous T2 35S-Xa47-JG30 plants and leaves at seedling stage. (**E-H**) JG30-WT and homozygous T2 35S-Xa47-JG30 plants and leaves at mature stage.

## Supplementary Table

| Table 1 Information of *Xoo* strains tested | | |
| --- | --- | --- |
| Code | Race/pathotype | Origin |
| YN18 (C1) | Chinese pathotype I | Yunnan, China |
| YN1 (C2) | Chinese pathotype II | Yunnan, China |
| GD414 (C3) | Chinese pathotype III | Guangdong, China |
| HEN11 (C4) | Chinese pathotype IV | Henan, China |
| ScYc-b (C5) | Chinese pathotype V | Yunnan, China |
| YN7 (C6) | Chinese pathotype VI | Yunnan, China |
| JS49-6 (C7) | Chinese pathotype VII | Jiangsu, China |
| FuJ (C8) | Chinese pathotype VIII | Fujian, China |
| YN24 (C9) | Chinese pathotype Ⅸ | Yunnan, China |
| PXO99 | Philippines race 6 (P6) | Philippines |
| PB | Mutants of P6 | Philippines |

Table 2 Detection of *Xa47* genotype in 100 ILs

| Cultivars/lines | Germplasm type | Genotype |
| --- | --- | --- |
| L2 | ILs | S |
| L3 | ILs | S |
| L4 | ILs | S |
| L9 | ILs | S |
| L13 | ILs | S |
| L15 | ILs | S |
| L24 | ILs | S |
| L30 | ILs | S |
| L34 | ILs | S |
| L35 | ILs | S |
| L42 | ILs | S |
| L44 | ILs | S |
| L46 | ILs | S |
| L49 | ILs | S |
| L54 | ILs | S |
| L55 | ILs | S |
| L57 | ILs | S |
| L62 | ILs | S |
| L64 | ILs | S |
| L70 | ILs | S |
| L73 | ILs | S |
| L74 | ILs | S |
| L75 | ILs | S |
| L76 | ILs | S |
| L80 | ILs | S |
| L83 | ILs | S |
| L85 | ILs | S |
| L92 | ILs | S |
| L103 | ILs | S |
| L110 | ILs | S |
| L111 | ILs | S |
| L127 | ILs | S |
| L129 | ILs | S |
| L130 | ILs | S |
| L131 | ILs | S |
| L137 | ILs | S |
| L141 | ILs | S |
| L165 | ILs | S |
| L167 | ILs | S |
| L168 | ILs | S |
| L188 | ILs | S |
| L189 | ILs | S |
| L194 | ILs | S |
| L195 | ILs | S |
| L196 | ILs | S |
| L197 | ILs | S |
| L198 | ILs | S |
| L201 | ILs | S |
| L203 | ILs | S |
| L205 | ILs | S |
| L206 | ILs | S |
| L233 | ILs | S |
| L240 | ILs | S |
| L244 | ILs | S |
| L246 | ILs | S |
| L280 | ILs | S |
| L287 | ILs | S |
| L291 | ILs | S |
| L292 | ILs | S |
| L294 | ILs | S |
| L300 | ILs | S |
| L313 | ILs | S |
| L317 | ILs | S |
| L321 | ILs | S |
| L322 | ILs | S |
| L325 | ILs | S |
| L331 | ILs | S |
| L333 | ILs | S |
| L336 | ILs | S |
| L337 | ILs | S |
| L345 | ILs | S |
| L351 | ILs | S |
| L353 | ILs | S |
| L354 | ILs | S |
| P357 | ILs | S |
| X1 | ILs | S |
| X11 | ILs | S |
| X13 | ILs | S |
| X14 | ILs | S |
| L17 | ILs | R |
| L20 | ILs | R |
| L27 | ILs | R |
| L250 | ILs | R |
| L18 | ILs | R |
| L26 | ILs | R |
| L50 | ILs | R |
| L61 | ILs | R |
| L66 | ILs | R |
| L102 | ILs | R |
| L105 | ILs | R |
| L113 | ILs | R |
| L114 | ILs | R |
| L116 | ILs | R |
| L117 | ILs | R |
| L119 | ILs | R |
| L126 | ILs | R |
| L156 | ILs | R |
| L159 | ILs | R |
| G252 | ILs | R |

Note: S and R represent *xa47* and *Xa47* genotypes, respectively.

Table 3 Detection of *Xa47* genotype in 80 Yunnan rice landraces

| Cultivars/lines | Accession | Origin | Subgroup | Genotype |
| --- | --- | --- | --- | --- |
| YN-1 | Yuxihonggu | Yongde | japonica | S |
| YN-2 | Honggu | Xishuangbanna | japonica | S |
| YN-3 | Xiaohungnuo | Honghe | japonica | S |
| YN-4 | Jiudigu | Puer | japonica | S |
| YN-5 | Mazhanuo | Honghe | japonica | S |
| YN-6 | Mujinuo | Honghe | japonica | S |
| YN-7 | Fanhaopi | Lincang | indica | S |
| YN-8 | Nuogu | Dehong | japonica | S |
| YN-9 | Babaomi | Wenshan | indica | S |
| YN-10 | Yingnuo | Xishuangbanna | indica | S |
| YN-11 | Wujugu | Dehong | indica | S |
| YN-12 | Honggganmazhagu | Wenshan | indica | S |
| YN-13 | Haonuoliang | Dehong | indica | S |
| YN-14 | Xiaobaimi | Yuxi | indica | S |
| YN-15 | Baikehongmi | Yuxi | indica | S |
| YN-16 | Lizihong | Yuxi | japonica | S |
| YN-17 | Ashugu | Puer | indica | S |
| YN-18 | Wulixiang | Wenshan | japonica | S |
| YN-19 | Haogan | Puer | indica | S |
| YN-20 | Sanbaibang | Zhaotong | japonica | S |
| YN-21 | Mazhagu | Puer | indica | S |
| YN-22 | Wuming | Dali | japonica | S |
| YN-23 | Danuogu | Qujing | japonica | S |
| YN-24 | Xiaowuju | Puer | japonica | S |
| YN-25 | Niuweituo | Zhaotong | japonica | S |
| YN-26 | Luoge | Honghe | indica | S |
| YN-27 | Daheilengshugu | Puer | japonica | S |
| YN-28 | Hangpinuo | Baoshan | japonica | S |
| YN-29 | Zalve | Nujiang | japonica | S |
| YN-30 | Mangjiangu | Puer | indica | S |
| YN-31 | Bendifangu | Zhaotong | indica | S |
| YN-32 | Zhuyajiugu | Zhaotong | japonica | S |
| YN-33 | Baijiugu | Zhaotong | japonica | S |
| YN-34 | Maxiangu | Lijiang | japonica | S |
| YN-35 | Yumixiang | Zhaotong | indica | S |
| YN-36 | Bendihonggu | Nujiang | japonica | S |
| YN-37 | Luoluogu | Diqing | japonica | S |
| YN-38 | Dabagu | Dehong | japonica | S |
| YN-39 | Baiyunzhan | Zhaotong | japonica | S |
| YN-40 | Jiegunuo | Yuxi | indica | S |
| YN-41 | Ganzhagu | Baoshan | japonica | S |
| YN-42 | Huangsinuogu | Zhaotong | japonica | S |
| YN-43 | Erbaikejiugu | Zhaotong | japonica | S |
| YN-44 | Magu | Baoshan | japonica | S |
| YN-45 | Gaonuogu | Zhaotong | japonica | S |
| YN-46 | Dananzhan | Zhaotong | indica | S |
| YN-47 | Dadiaogu | Zhaotong | indica | S |
| YN-48 | Huangjiaonuozaogu | Zhaotong | indica | S |
| YN-49 | Hongjiugu | Zhaotong | japonica | S |
| YN-50 | Mazhan | Zhaotong | japonica | S |
| YN-51 | Xiaozaogu | Honghe | indica | S |
| YN-52 | Babagu | Yuxi | japonica | S |
| YN-53 | Huangkenuo | Baoshan | indica | S |
| YN-54 | Dabaigu | Zhaotong | indica | S |
| YN-55 | Damazhan | Lijiang | indica | S |
| YN-56 | Xuan6hao | Lijiang | japonica | S |
| YN-57 | Qishixiang | Zhaotong | japonica | S |
| YN-58 | Wujiaozhan | Zhaotong | indica | S |
| YN-59 | nangaogu | Lincang | indica | S |
| YN-60 | Haomoya | Dehong | indica | S |
| YN-61 | Zaogu | Puer | japonica | S |
| YN-62 | Haogelao | Puer | japonica | S |
| YN-63 | Haohuan | Lincang | japonica | S |
| YN-64 | Heigu | Lincang | japonica | S |
| YN-65 | WumingA | Dehong | indica | S |
| YN-66 | Dahuangpinuo | Lincang | japonica | S |
| YN-67 | Huangxiannuo | Chuxiong | japonica | S |
| YN-68 | Yanzhidiao | Chuxiong | japonica | S |
| YN-69 | Landigu | Puer | japonica | S |
| YN-70 | Heijianggu | Baoshan | japonica | S |
| YN-71 | Haolaiqing | Lincang | indica | S |
| YN-72 | Jinbaoyin | Honghe | japonica | S |
| YN-73 | Huangxiangnuo | Honghe | japonica | R |
| YN-74 | Laoyaling | Lincang | japonica | R |
| YN-75 | Changmanggu | Yuxi | japonica | R |
| YN-76 | Hangu | Yuxi | japonica | R |
| YN-77 | Dabainuo | Baoshan | japonica | R |
| YN-78 | Xilandigu | Baoshan | indica | R |
| YN-79 | Haojiege | Xishuangbanna | japonica | R |
| YN-80 | Shuibaishidian | Lincang | indica | R |

Note: S and R represent *xa47* and *Xa47* genotypes, respectively.

| Table 4 Primer information used in this study | | | | |
| --- | --- | --- | --- | --- |
| Primer name | Primer sequence 5'-3' | | Purpose | |
| Xa47-12-F | | TGGTGCCTATACCTTCATTG | | Gene clone |
| Xa47-12-R | | AATTCGTCATGTTCTACTAGC | |  |
| Hxjy-1-F | | GACTGTTCCCTCTTGGATAC | | Molecular marker |
| Hxjy-1-R | | GTCGTGAAGAAAGAAGCAAG | |  |
| Xa47-CDS-F | | ATGACGGGGGAGGAAGTC | | CDS amplification |
| Xa47-CDS-R | | ATGTATGATGCATGTCACG | |  |
| Xa47-GFP-F | | gagaacacgggggactctagaATGACGGGGGAGGAAG | | XA47::GFP vector construction |
| Xa47-GFP-R | | gctcaccataagcttgtcgacATACCATATACATGTATGATGCATGTCAC | |  |
| TG-F | | ggcaCAAGGTGCCGGAAAAAAGAA | | pOs-Xa47-sgRNA vector construction |
| TG-R | | aaacTTCTTTTTTCCGGCACCTTG | |  |
| Xa47-35S-F | | acgggggactcttgaccatggATGACGGGGGAGGAAGTC | | 35S::Xa47 vector construction |
| Xa47-35S-R | | gggaaattcgagctggtcaccATGTATGATGCATGTCACG | |  |
| Actin-F | | CAGGCCGTCCTCTCTCTGTA | | qRT-PCR |
| Actin-R | | AAGGATAGCATGGGGGAGAG | |  |
| qRT-Xa47-F | | TCCACCCTCCGGAATAAGGT | | qRT-PCR |
| qRT-Xa47-R | | TGGAATTGCTGCTCTGTCGT | |  |
| OsNPRl-F | | AAACCGGATCAGTTTCATCA | | qRT-PCR |
| OsNPRl-R | | AAGAACACTTAGCTCGGATGAC | |  |
| OsPR1a-F | | TTCATCACCTGCAACTACTCG | | qRT-PCR |
| OsPR1a-R | | TGCATAAACACGTAGCATAGCAT | |  |
| OsPRl0-F | | CACCATCTACACCATGAAGC | | qRT-PCR |
| OsPRl0-R | | AGCACATCCGACTTTAGGAC | |  |
